# Progress Toward Measles Elimination — Worldwide, 2000–2022

**DOI:** 10.15585/mmwr.mm7246a3

**Published:** 2023-11-17

**Authors:** Anna A. Minta, Matt Ferrari, Sebastien Antoni, Allison Portnoy, Alyssa Sbarra, Brian Lambert, Cynthia Hatcher, Christopher H. Hsu, Lee Lee Ho, Claudia Steulet, Marta Gacic-Dobo, Paul A. Rota, Mick N. Mulders, Anindya Sekhar Bose, William Perea Caro, Patrick O’Connor, Natasha S. Crowcroft

**Affiliations:** ^1^Immunization, Vaccines, and Biologicals, World Health Organization, Geneva, Switzerland; ^2^Center for Infectious Disease Dynamics, Pennsylvania State University, University Park, Pennsylvania; ^3^Department of Global Health, Boston University School of Public Health, Boston, Massachusetts; ^4^Department of Infectious Disease Epidemiology, London School of Hygiene & Tropical Medicine, London, United Kingdom; ^5^Global Immunization Division, Center for Global Health, CDC; ^6^Division of Viral Diseases, National Center for Immunization and Respiratory Diseases, CDC.

**Summary**

**What is already known about this topic?**

Global coverage with measles-containing vaccine (MCV) declined during the COVID-19 pandemic to the lowest levels since 2008, and measles surveillance was suboptimal.

**What is added by this report?**

During 2000–2022, estimated measles vaccination prevented approximately 57 million deaths worldwide. However, millions of children missed vaccinations during the COVID-19 pandemic, resulting in an 18% increase in estimated measles cases and a 43% increase in estimated measles deaths in 2022 compared with 2021. Large or disruptive outbreaks were reported in 37 countries. Measles surveillance remains suboptimal.

**What are the implications for public health practice?**

To continue progress toward measles elimination, all children should receive 2 MCV doses to address pandemic-related immunity gaps and measles surveillance should be strengthened.

## Abstract

Measles is a highly contagious, vaccine-preventable disease that requires high population immunity for transmission to be interrupted. All six World Health Organization regions have committed to eliminating measles; however, no region has achieved and sustained measles elimination. This report describes measles elimination progress during 2000–2022. During 2000–2019, estimated coverage worldwide with the first dose of measles-containing vaccine (MCV) increased from 72% to 86%, then declined to 81% in 2021 during the COVID-19 pandemic, representing the lowest coverage since 2008. In 2022, first-dose MCV coverage increased to 83%. Only one half (72) of 144 countries reporting measles cases achieved the measles surveillance indicator target of two or more discarded cases per 100,000 population in 2022. During 2021–2022, estimated measles cases increased 18%, from 7,802,000 to 9,232,300, and the number of countries experiencing large or disruptive outbreaks increased from 22 to 37. Estimated measles deaths increased 43% during 2021–2022, from 95,000 to 136,200. Nonetheless, an estimated 57 million measles deaths were averted by vaccination during 2000–2022. In 2022, measles vaccination coverage and global surveillance showed some recovery from the COVID-19 pandemic setbacks; however, coverage declined in low-income countries, and globally, years of suboptimal immunization coverage left millions of children unprotected. Urgent reversal of coverage setbacks experienced during the COVID-19 pandemic can be accomplished by renewing efforts to vaccinate all children with 2 MCV doses and strengthening surveillance, thereby preventing outbreaks and accelerating progress toward measles elimination.

## Introduction

Measles is a highly contagious, vaccine-preventable disease that requires high population immunity for transmission to be interrupted. All six World Health Organization (WHO) regions have committed to eliminating measles*; however, no region has achieved and sustained measles elimination. The Immunization Agenda 2030 (IA2030)^†^ includes measles elimination as a core indicator of impact. IA2030 highlights the importance of ensuring rigorous measles surveillance systems to identify immunity gaps, and of achieving equitable 95% coverage with 2 timely childhood doses of measles-containing vaccine (MCV). Because measles is highly infectious, failures of routine immunization services to reach children are rapidly revealed by the occurrence of outbreaks primarily affecting unvaccinated children. Thus, measles infections act as a tracer of the ability of the health system to deliver essential vaccines in childhood. This report describes progress toward measles elimination during 2000–2022, including immunization activities, assessment of surveillance performance, numbers of measles cases, estimates of the number of measles cases and deaths, and elimination verification status, and updates a previous report (*1*).

## Methods

### Immunization and Surveillance Data Collection and Analysis

WHO and UNICEF estimate coverage with the first and second MCV doses (MCV1 and MCV2, respectively) delivered through routine immunization services^§^ for all countries, using annual administrative coverage data (the number of vaccine doses administered divided by the estimated target population), national coverage estimates,^¶^ and vaccination coverage surveys. Countries report the annual number of incident measles cases to WHO and UNICEF, using the Joint Reporting Form, and these data are used to calculate measles incidence.** The Global Measles and Rubella Laboratory Network (GMRLN) consists of 743 laboratories that support measles and rubella surveillance by providing quality-controlled laboratory testing to detect measles-specific immunoglobulin M in serum specimens and to perform genotyping of measles virus from clinical specimens (*2*). 

### Modeling Estimates

A previously described model for estimating measles cases and deaths was updated with 2022 measles data and United Nations 2000–2022 population estimates^††^ (*3*). Data on case fatality rates from a publicly available statistical package (measlesCFR)^§§^ were used in the model to calculate estimates of measles mortality, based on previously published methodology (*4*). These activities were reviewed by CDC, deemed not research, and were conducted consistent with applicable federal law and CDC policy.^¶¶^

## Results

### Immunization Activities

During the first 2 decades of the millennium (2000–2019), estimated MCV1 coverage worldwide increased from 72% to 86%, then declined to 83% in 2020 during the COVID-19 pandemic, and declined further to 81% in 2021 (Table 1). Coverage in all regions declined during 2019–2021. In 2022, global coverage increased to 83%, and increased in all regions except in the Americas and the European Region. Regional coverage remained below 2019 levels in all regions except the Eastern Mediterranean Region. During 2019–2021, MCV1 coverage in low-income countries fell from 71% to 67%, then to 66% in 2022 (Supplementary Table 1, https://stacks.cdc.gov/view/cdc/135223).

**TABLE 1:** Estimates of regional immunization coverage with the first and second doses of measles-containing vaccine administered through routine immunization services, reported measles cases, and measles incidence, by World Health Organization region — worldwide, 2000–2022

WHO region/yr (no. of countries in region)	Percentage				No. of reported measles cases^§^ (% of total cases)	Measles incidence ^§,¶,^**
	MCV1 coverage*	Countries with ≥95% MCV1 coverage^†^	MCV2 coverage*	Reporting countries with <5 measles cases per 1 million population^§,¶^		
**Total (all regions)**						
**2000 (191)**	**72**	**28**	**17**	**33**	**853,479 (100.0)**	**145.3**
**2010 (193)**	**84**	**45**	**42**	**59**	**343,806 (100.0)**	**49.7**
**2016 (194)**	**85**	**42**	**67**	**64**	**132,490 (100.0)**	**18.1**
**2019 (194)**	**86**	**44**	**71**	**44**	**873,022 (100.0)**	**119.5**
**2020 (194)**	**83**	**30**	**72**	**57**	**159,073 (100.0)**	**21.3**
**2021 (194)**	**81**	**29**	**71**	**68**	**123,171 (100.0)**	**16.7**
**2022 (194)**	**83**	**34**	**74**	**58**	**205,153 (100.0)**	**28.8**
**African**						
2000 (46)	53	2	5	6	**520,102 (60.9)**	832.3
2010 (46)	72	17	5	30	**199,174 (57.9)**	231.5
2016 (47)	69	17	22	49	**36,269 (27.4)**	36.5
2019 (47)	71	13	33	34	**618,595 (70.9)**	559.8
2020 (47)	70	6	40	30	**115,369 (72.5)**	106.3
2021 (47)	68	4	41	34	**88,789 (72.1)**	81.9
2022 (47)	69	11	45	21	**97,237 (47.4)**	81.6
**Americas**						
2000 (35)	93	40	65	89	**1,754 (0.2)**	2.1
2010 (35)	93	49	67	100	**247 (0.1)**	0.3
2016 (35)	92	46	80	97	**97 (0.1)**	0.1
2019 (35)	87	40	73	89	**21,971 (2.5)**	32.3
2020 (35)	85	20	72	97	**9,996 (6.3)**	9.8
2021 (35)	85	14	75	97	**682 (0.6)**	0.7
2022 (35)	84	17	76	86	**47 (—)**	0.1
**Eastern Mediterranean**						
2000 (21)	71	29	27	14	**38,592 (4.5)**	86.9
2010 (21)	76	52	52	38	**10,072 (2.9)**	16.5
2016 (21)	82	57	73	52	**6,275 (4.7)**	9.5
2019 (21)	83	48	76	38	**18,458 (2.1)**	26.4
2020 (21)	83	38	77	48	**6,769 (4.3)**	10.3
2021 (21)	82	43	77	52	**26,089 (21.2)**	39.8
2022 (21)	83	48	78	38	**56,401 (27.5)**	82.4
**European**						
2000 (52)	91	45	48	38	**37,421 (4.4)**	50.0
2010 (53)	94	64	80	68	**30,625 (8.9)**	34.2
2016 (53)	93	49	88	77	**4,440 (3.4)**	5.2
2019 (53)	96	60	92	30	**106,130 (12.2)**	116.6
2020 (53)	94	43	91	70	**10,945 (6.9)**	13.5
2021 (53)	94	47	92	92	**99 (0.1)**	0.1
2022 (53)	93	49	91	85	**825 (0.4)**	1.1
**South-East Asia**						
2000 (10)	62	18	3	0	**78,558 (9.2)**	50.5
2010 (11)	83	45	15	36	**54,228 (15.8)**	29.7
2016 (11)	89	55	75	27	**27,530 (20.8)**	14.0
2019 (11)	94	64	83	27	**29,389 (3.4)**	14.7
2020 (11)	88	45	80	45	**9,389 (5.9)**	4.8
2021 (11)	86	45	78	55	**6,448 (5.2)**	3.3
2022 (11)	92	55	85	64	**49,201 (24.0)**	23.8
**Western Pacific**						
2000 (27)	85	30	2	26	**177,052 (20.7)**	106.0
2010 (27)	97	44	87	63	**49,460 (14.4)**	27.5
2016 (27)	96	52	93	48	**57,879 (43.7)**	30.9
2019 (27)	95	59	93	41	**78,479 (9.0)**	41.0
2020 (27)	94	44	93	37	**6,605 (4.2)**	3.5
2021 (27)	90	41	91	56	**1,064 (0.9)**	0.6
2022 (27)	92	44	91	44	**1,442 (0.7)**	0.8

**Abbreviations:** MCV1 = first dose of measles-containing vaccine; MCV2 = second dose of measles-containing vaccine; WHO = World Health Organization.

* https://immunizationdata.who.int/pages/coverage/mcv.html (Accessed July 31, 2023).

^†^ Denominator is the number of WHO member states.

^§^ https://immunizationdata.who.int/pages/incidence/measles.html (Accessed August 7, 2023).

^¶^ Population data from United Nations Department of Economic and Social Affairs, Population Division, 2022. Any country not reporting measles cases for that year was removed from both the numerator and denominator in calculating incidence.

** Cases per 1 million population.

Among the 194 WHO countries, 65 (34%) achieved ≥95% MCV1 coverage in 2022. In 2022, the 21.9 million infants who did not receive MCV1 through routine immunization services represented a decrease of 2.5 million (10%) compared with 2021, and a 2.7 million increase compared with 2019. The 10 countries with the highest number of infants who did not receive MCV1 were Nigeria (3 million), Democratic Republic of the Congo (1.8 million), Ethiopia (1.7 million), India (1.1 million), Pakistan (1.1. million), Angola (0.8 million), Philippines (0.8 million), Indonesia (0.7 million), Brazil (0.5 million), and Madagascar (0.5 million). These 10 countries accounted for 55% of all children worldwide who did not receive MCV1. The top nine countries also had the highest number of children who had not received MCV1 in 2021 (Madagascar replaced Tanzania as the 10th country in 2022).

Estimated MCV2 coverage increased from 17% in 2000 to 74% in 2022,*** largely as a result of vaccine introductions; however, 11 million children did not receive MCV2 through routine immunization in 2022. The number of countries offering MCV2 increased by 98%, from 95 (49%) in 2000 to 188 (97%) in 2022. Six countries (Chad, Democratic Republic of the Congo, Guinea, Guinea-Bissau, Somalia, and Uganda) introduced MCV2 in 2022, and six countries (Benin, Central African Republic, Gabon, Mauritania, South Sudan, and Vanuatu) have yet to introduce MCV2.^†††^

Approximately 115 million persons received MCV through supplementary immunization activities (SIAs)^§§§^ in 44 countries in 2022, and an additional 16 million received MCV during measles outbreak response activities. Among 41 MCV campaigns delayed because of the COVID-19 pandemic, 35 (85%) in 29 countries had been conducted by the end of December 2022.

### Surveillance Performance and Reported Measles Incidence

Among the 144 (74%) countries that reported discarded cases^¶¶¶^ in 2022, 72 (50%) achieved the measles surveillance sensitivity indicator target of two or more discarded cases per 100,000 population, compared with 47 (35%) of 135 countries reporting in 2021, 45 (31%) of 143 countries reporting in 2020, and 46 (32%) of 144 countries reporting in 2019. In 2022, GMRLN laboratories received 273,080 specimens for measles testing compared with 139,319 in 2021, 121,257 in 2020, and 282,020 in 2019.

During 2000–2016, the number of reported measles cases declined 85%, from 853,479 to 132,490, corresponding to an 88% decrease in incidence, from 145 cases to 18 cases per 1 million population. During 2019, the number of reported cases (837,922) and reported measles incidence (120 per million) increased more than fivefold compared with 2016. The number of cases then declined to 123,171 in 2021 (incidence of 17 per million) but increased 67% to 205,153 in 2022; incidence increased 71% from 17 to 29 per 1 million population from 2021 to 2022.

In 2022, 37 countries in four WHO regions were affected by large or disruptive measles outbreaks,**** an increase of 68% compared with 22 countries in two regions the preceding year. Among these 2022 outbreaks, 28 of 37 (76%) occurred in countries in the African Region, six (16%) in the Eastern Mediterranean Region, two (5%) in the South-East Asia Region, and one (3%) in the European Region (Supplementary Table 2, https://stacks.cdc.gov/view/cdc/135224). Overall, 31 of 72 countries (43%) achieving the measles surveillance sensitivity indicator target had large or disruptive measles outbreaks during 2022.

Genotypes detected from measles cases^††††^ were reported by 35 (33%) of the 105 countries reporting at least one measles case in 2022, compared with 22 (27%) of 82 such countries in 2021. As a result of global elimination activities, the number of genotypes detected has been decreasing over time, from 13 in 2002 to two in 2021 and 2022. A total of 800 sequences were reported in 2021, among which 608 (76%) were genotype B3 and 192 (24%) were genotype D8; among 1,470 reported sequences in 2022, 772 (53%) were genotype D8 and 698 (47%) were genotype B3.

### Measles Cases and Mortality Estimates

On the basis of the revised model for estimating measles cases and deaths and 2022 data, the estimated number of measles cases decreased 75%, from an estimated 36,463,000 in 2000 to 9,232,300 in 2022; the estimated annual number of measles deaths decreased 82%, from 772,900 in 2000 to 136,200 in 2022 (Table 2). The estimated number of cases increased 18% and deaths increased 43% in 2022 compared with an estimated 7,802,000 cases and estimated 95,000 deaths in 2021. During 2000–2022, measles vaccination prevented an estimated 57 million deaths globally, compared with no vaccination (Figure).

**TABLE 2:** Estimated number of measles cases and deaths,* by World Health Organization region — worldwide, 2000 and 2022

WHO region/yr (no. of countries in region)	Estimated no. (95% CI)		Measles 2000–2022	
	Measles cases	Measles deaths	% Estimated reduction in mortality	Cumulative no. of deaths averted by vaccination
**Total (all regions)**				
**2000 (191)**	**36,462,747** **(27,651,303–49,998,176)**	**772,854** **(580,969–1,064,580)**	**82**	**57,193,384**
**2022 (194)**	**9,232,288** **(6,163,724–14,176,076)**	**136,216** **(97,058–190,234)**		
**African**				
2000 (46)	11,789,801 (8,663,242–16,377,945)	352,856 (265,311–482,300)	76	19,503,394
2022 (47)	5,138,698 (3,706,922–6,748,362)	85,417 (59,449–117,685)		
**Americas**				
2000 (35)	8,770 (4,385–35,080)	3	91	6,078,056
2022 (35)	825 (413–3,300)	1^†^		
**Eastern Mediterranean**				
2000 (21)	4,183,126 (2,699,324–7,632,325)	134,250 (94,319–222,647)	71	9,109,711
2022 (21)	1,193,257 (827,241–1,928,555)	39,656 (30,318–53,601)		
**European**				
2000 (52)	866,396 (453,826–1,504,507)	3,584 (2,206–5,503)	98	1,449,774
2022 (53)	63,707 (19,753–167,892)	70 (20–201)		
**South-East Asia**				
2000 (10)	13,943,036 (11,008,470–17,255,557)	255,133 (197,243–321,745)	96	16,362,284
2022 (11)	1,896,917 (1,322,645–2,910,260)	9,542 (6,839–14,248)		
**Western Pacific**				
2000 (27)	5,671,618 (4,822,056–7,192,761)	27,028 (21,889–32,373)	94	4,690,166
2022 (27)	938,883 (286,751–2,417,707)	1,531 (432–4,498)		

**Abbreviation:** WHO = World Health Organization.

* The measles mortality model used to generate estimated measles cases and deaths is rerun each year using the new and revised annual WHO/UNICEF estimates of national immunization coverage data, as well as updated surveillance data.

^†^ Estimated measles mortality rounded to 1.

**FIGURE:**
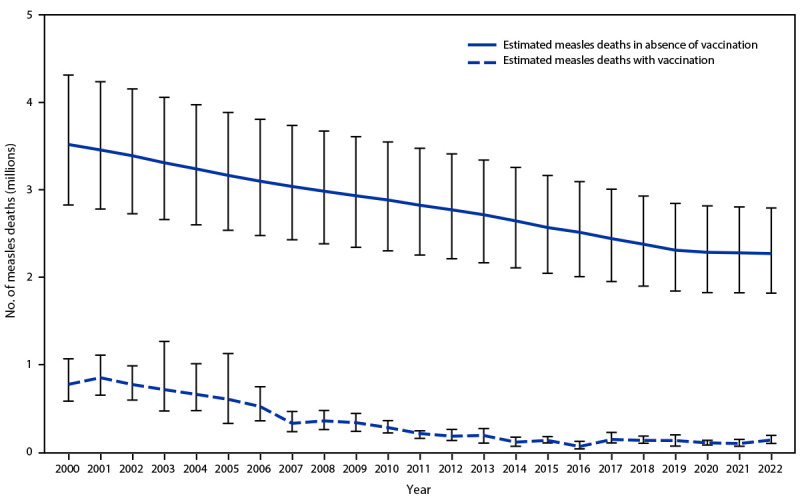
Estimated number of annual measles deaths with measles vaccination and in the absence of measles vaccination — worldwide, 2000–2022*^,^^†^ * With 95% CIs indicated by error bars. ^†^ Deaths prevented by vaccination are estimated by the area between estimated deaths with vaccination and those without vaccination. A cumulative total of 57 million deaths were estimated to have been prevented by vaccination during 2000–2022.

### Regional Verification of Measles Elimination

By the end of 2022, 83 countries (43% of all countries) had been verified by independent regional commissions to have achieved or maintained measles elimination, although no WHO region had achieved and sustained elimination, and no African Region country had yet been verified to have eliminated measles (Supplementary Table 3, https://stacks.cdc.gov/view/cdc/135225). WHO’s Region of the Americas achieved verification of measles elimination in 2016; however, endemic measles transmission was reestablished in Brazil and Venezuela. Since 2016, endemic transmission has been reestablished in seven other countries (Albania, Czechia, Lithuania, Slovakia, and Uzbekistan in the European Region; and Cambodia and Mongolia in the Western Pacific Region) that had previously achieved verification of measles elimination. The United Kingdom was verified to have achieved measles elimination in 2021, after reestablishment of transmission in 2018 after initial verification of elimination in 2016.

## Discussion

Globally, the decline in MCV coverage during the COVID-19 pandemic has shown some recovery in 2022; however, the trend is not consistent across regions, and no region has achieved the recommended 95% coverage with 2 doses of MCV necessary for elimination (*5*). Vaccination coverage declined most in low-income countries where risk for death from measles is likely highest. SIAs provide essential means to decrease immunity gaps and vaccinate children who missed MCV doses during routine immunization activities.^§§§§^ Immunization programs will need to accelerate immunization program recovery to close these immunity gaps and reduce disease incidence.

Although measles surveillance performance has improved, half of all countries did not meet the surveillance sensitivity target indicator in 2022. In addition, the discarded case rate might have increased because of increased testing of suspected measles cases during outbreaks in 2022 rather than an actual improvement in surveillance performance. Measles incidence declined during 2020 and 2021, potentially because of decreased virus transmission related to COVID-19 mitigation measures, surveillance disruptions, and immunity acquired through high rates of infection during the global measles resurgence during 2017–2019. From 2021 to 2022, reported measles cases increased 67% globally, and the number of countries experiencing large or disruptive outbreaks increased by 68% as COVID-19 mitigation measures were lifted, surveillance improved, and declining MCV coverage left millions of children unprotected from measles.

### Limitations

The findings in this report are subject to at least three limitations. First, vaccination coverage might be affected by data quality issues, leading to inaccurate estimations. Second, the number of specimens submitted for genotyping represents a small proportion of measles cases, so the distribution of genotypes presented might not reflect the global distribution. Finally, the output from modeling estimates is dependent on the data input into the model and is thus subject to some uncertainty.

### Implications for Public Health Practice

Since 2000, measles vaccination has averted an estimated 57 million deaths worldwide; however, the COVID-19 pandemic disrupted global vaccination activities, which in 2021 resulted in the lowest MCV1 coverage levels since 2008. Measles immunization coverage began improving in 2022 but has not reached 2019 prepandemic levels and remains far from the ≥95% 2-dose MCV coverage target. Approximately 21.9 million children did not receive any dose of MCV in 2022, leaving a large population susceptible to measles infection and outbreaks. Only 36 (19%) of 194 countries exceeded 2019 coverage levels by 2022, resulting in an accumulation of susceptible children born during the pandemic years. Global measles surveillance, after setbacks during the COVID-19 pandemic, still needs improvement. The Measles and Rubella Strategic Framework,^¶¶¶¶^ which aligns with IA2030, includes strategies that countries can draw upon to improve routine immunization, prioritize comprehensive surveillance, and employ data-driven decision-making to strengthen national and subnational capacity for outbreak preparedness and response and address immunity gaps to reach all children. It is critical that all countries and global partners work to accelerate the recovery of vaccination and surveillance programs toward the end goal of regional measles elimination.

## Footnotes

* Measles elimination is defined as the absence of endemic measles virus transmission in a region or other defined geographic area for ≥12 months in the presence of a high-quality surveillance system that meets the targets of key performance indicators.

†
https://www.who.int/immunization/immunization_agenda_2030/en/

§ Calculated for MCV1, among children aged 1 year or, if MCV1 is given at age ≥1 year, among children aged 24 months. Calculated for MCV2 among children at the recommended age for the administration of MCV2, according to the national immunization schedule. https://www.who.int/teams/immunization-vaccines-and-biologicals/immunization-analysis-and-insights/global-monitoring/immunization-coverage/who-unicef-estimates-of-national-immunization-coverage (Accessed July 31, 2023).

¶ Estimates based on administrative data and any other available information on factors affecting immunization coverage, including private or nongovernmental organization sector contributions to immunization, difficulties with demographic data, and incomplete reporting.

** To calculate incidence, only countries that reported data are included in the numerator and denominator. Countries do not provide WHO with their reasons for not reporting measles cases. https://immunizationdata.who.int/pages/incidence/measles.html (Accessed August 7, 2023).

†† State-space model of unobserved measles incidence during 2000–2022 generated using the following inputs from all member countries: 1) total annual reported measles cases; 2) annual MCV1 coverage from WHO and UNICEF estimates of national immunization coverage (WUENIC); 3) annual MCV2 coverage from WUENIC; 4) annual SIAs, with coverage and age targets (subnational SIAs are discounted by the proportion of the total population targeted); 5) total annual population size; 6) total annual births; and 7) list of all countries and years for which reporting was enhanced.

§§ The measlesCFR model (https://github.com/Measles-Case-Fatality-Ratio-Estimation/measlesCFR) fitted the reported case fatality ratios from the systematic review as a function of the following covariates: 1) gross domestic product per capita, 2) HIV prevalence, 3) maternal education, 4) MCV1 coverage, 5) proportion urban, 6) total fertility rate, 7) mortality rate among children aged <5 years, 8) prevalence of vitamin A deficiency, 9) war and terrorism mortality rate, 10) wasting (weight-for-height more than 1 standard deviation below the reference median) prevalence (https://www.healthdata.org/results/gbd_summaries/2019/child-wasting-level-4-risk), and 11) measles incidence. Annual measles incidence for each country and year was based on this fitted state-space model. High income countries were excluded from this analysis.

¶¶ 45 C.F.R. part 46.102(l)(2), 21 C.F.R. part 56; 42 U.S.C. Sect. 241(d); 5 U.S.C. Sect. 552a; 44 U.S.C. Sect. 3501 et seq.

***
https://immunizationdata.who.int/listing.html?topic=&location=

††† Data as of July 31, 2023. http://immunizationdata.who.int/pages/vaccine-intro-by-antigen/mcv2.html?ISO_3_CODE=&YEAR=

§§§ Measles SIAs are generally conducted using two target age ranges: 1) an initial catch-up SIA targets children aged 9 months–14 years, with the aim of eliminating susceptibility to measles in the general population, and 2) periodic follow-up SIAs are conducted nationwide every 2–4 years and target all children aged 9–59 months to eliminate any measles susceptibility that has accumulated in recent birth cohorts because of low MCV coverage and to protect the estimated 2%–5% of children who did not respond to MCV1. Countries can provide additional data to WHO, and data are updated retrospectively.

¶¶¶ A discarded measles case is defined as a suspected case that has been investigated and determined to be neither measles nor rubella by using either 1) laboratory testing in a proficient laboratory or 2) epidemiologic linkage to a laboratory-confirmed outbreak of a communicable disease that is not measles or rubella. The discarded case rate is used to measure the sensitivity of measles surveillance.

**** IA2030 global monitoring framework defines large or disruptive outbreaks as having ≥20 cases per 1 million population.

†††† Data as of August 23, 2023. https://who-gmrln.org/means2

§§§§ https://apps.who.int/iris/handle/10665/360891; https://apps.who.int/iris/handle/10665/340657

¶¶¶¶
https://www.who.int/publications/i/item/measles-and-rubella-strategic-framework-2021-2030
